# Imidazolinium and amidinium salts as Lewis acid organocatalysts

**DOI:** 10.3762/bjoc.8.205

**Published:** 2012-10-18

**Authors:** Oksana Sereda, Nicole Clemens, Tatjana Heckel, René Wilhelm

**Affiliations:** 1 Institute of Organic Chemistry, Clausthal University of Technology, 38678 Clausthal-Zellerfeld, Germany; 2 Department of Chemistry, University of Paderborn, 33098 Paderborn, Germany

**Keywords:** Diels–Alder, ionic liquids, organocatalysis, soft Lewis acids, thiiranes, thioesters

## Abstract

The application of imidazolinium and amidinium salts as soft Lewis acid organocatalysts is described. These salts were suitable catalysts for the activation of unsaturated thioesters in a Diels–Alder reaction and in the ring opening of thiiranes and epoxides. The products were isolated in good yields. The mild catalysts did not cause desulfurization of the products containing a thiol or thiocarbonyl group.

## Introduction

Salts with melting points below 100 °C are known as ionic liquids and are often used as novel solvents for reactions and electrochemical processes [1]. Several of these solvents can contribute to the research field of “green chemistry” [2]. The most common used ionic liquids are based on imidazolium cations. Next to their application in catalytic reactions [3–6], they are also capable of catalyzing reactions themselves, either in substoichiometric amounts or as reaction medium due to hydrogen-bond activation of the protons of the imidazolium cation [7–10] and other variables, such as π-orbital and charge–charge interactions [8–10].

Recently, we applied saturated imidazolium salts with an aryl substituent at the C(2) position as catalysts for the aza-Diels–Alder reaction [11–12]. These catalysts do not activate the substrate through hydrogen bonding, but instead, like other carbon-cation based catalysts, through their positive center [8–10,13–15], and belong also to the field of organocatalysis [10,16–17].

Taking the soft Lewis acidic character of imidazolinium salts into consideration, we were interested to apply these salts in a Diels–Alder reaction with ethyl crotonthioate as dienophile. Due to the low electronegativity of sulfur it would be difficult to catalyze this reaction with hydrogen-bond activation catalysts [18]. The utilization of the soft Lewis basic sulfur groups in this reaction is rare. Often mercury Lewis acids are applied [19–22] to activate the sulfur group and there is also the risk of desulfurization [23].

**Scheme 1:**
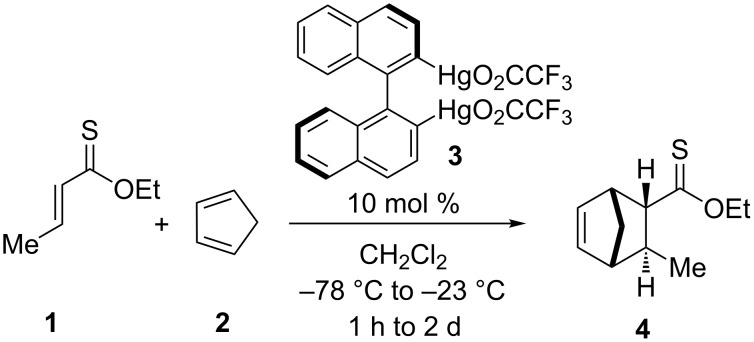
Diels–Alder reaction with ethyl crotonthioate (**1**) and cyclopentadiene (**2**).

The only known asymmetric Diels–Alder reaction with ethyl crotonthioate (**1**) and cyclopentadiene (**2**) in the presence of optically pure 2,2'-dimercurio-1,1'-binaphthyl compound **3** gave the product **4** in 44% yield with 58% ee (Scheme 1) [20].

## Results and Discussion

**Scheme 2:**
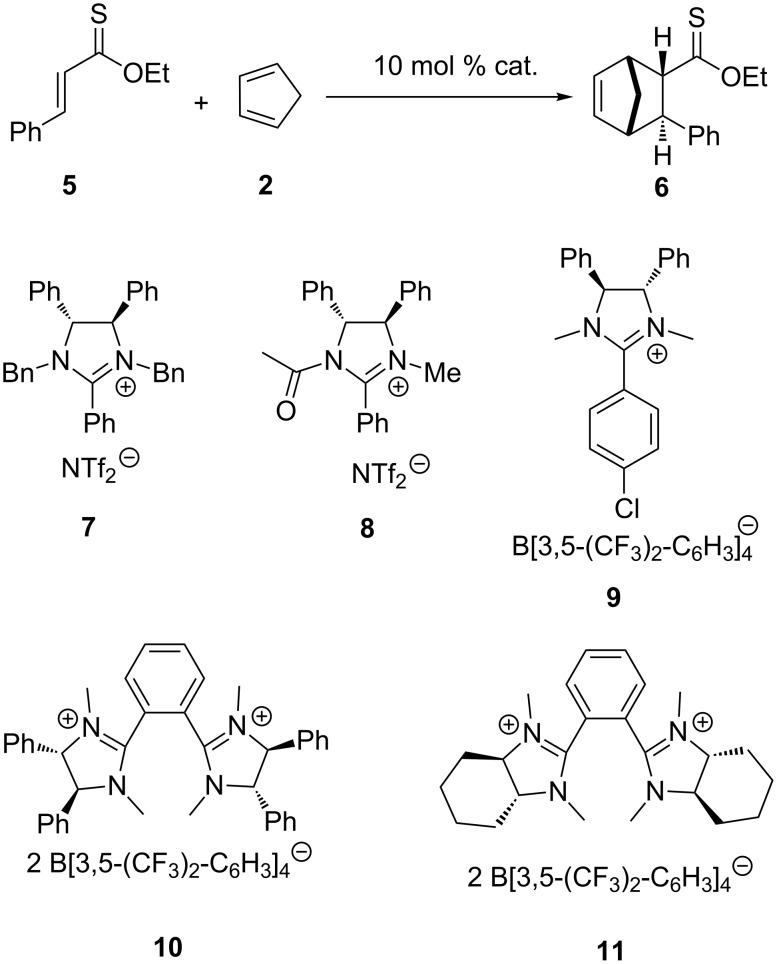
Diels–Alder reaction catalysed with imidazolinium salts.

Since product **4** has a low boiling point and proved difficult to purify on a small scale, the model reaction shown in Scheme 2 was evaluated. The α,β-unsaturated thioester **5** was prepared from ethyl cinnamate and 2,4-bis(4-methoxyphenyl)-1,3,2,4-dithiadiphosphetane-2,4-disulfide (Lawesson’s reagent) [22]. The cycloaddition of ethyl thionocinnamate (**5**) with 1.5 equiv of cyclopentadiene (**2**) was performed in different solvents, at varied temperatures, by using a broad range of catalysts, which are summarized in Table 1.

**Table 1:** Diels–Alder reaction with 10 mol % catalyst according to Scheme 2. Reactions with CH_2_Cl_2_ were carried out in a pressure vessel.

entry	solvent	catalyst	temp [°C]	time [d]	yield %^a^ (*endo*:*exo*)

1	CH_2_Cl_2_	Hg(OAc)_2_	−10–45	4	0
2	CH_2_Cl_2_	BF_3_^.^Et_2_O	−10–45	4	90 (99:1)
3	CH_2_Cl_2_	TMSOTf	−10–45	4	55 (99:1)
4	CH_2_Cl_2_	**7**	25–45	4	8 (13:1)
5	toluene	**7**	25–110	4	0
6	dioxane	**7**	25–110	4	6 (3:1)
7	CH_2_Cl_2_	**8**	25–45	4	5 (9:1)
8	CH_2_Cl_2_	**9**	0–45	4	12 (14:1)
9	CH_2_Cl_2_	**10**	−10–45	4	69 (99:1)
10	CH_2_Cl_2_	**11**	0–45	3	66 (99:1)

^a^Isolated yields.

First a few metal-based Lewis acids were applied in the reaction. BF_3_•Et_2_O is known to activate also sulfur carbonyl groups [24] and gave the product in 90% yield, while TMSOTf gave the product in 55% yield. In both cases the *endo* product was the major product. The reaction was followed by TLC and the temperature was gradually increased from –10 to 45 °C over 4 days in ca. 10 °C steps.

Next, imidazolinium salt **7** [25] was used in the reaction. The formation of the product was observed at 45 °C; however, the product was isolated only in a low yield of 8%. In toluene no reaction was observed even under reflux. In dioxane under reflux a low yield of 6% was obtained with a low *endo*/*exo* ratio of (3:1). Salt **8** [25] and **9** [25] gave similar poor results as salt **7** [25]. Finally, the bis-imidazolinium salt **10** [12] gave the product in 69% yield with an *endo*/*exo* ratio comparable to BF_3_•Et_2_O. The reaction started slowly at 10 °C. In addition, the similar salt **11** [12] gave the product in a comparable yield of 66%. Although in all cases the product was racemic, the results show that bis-imidazolinium salts can be applied as Lewis acid organocatalysts to activate a thiocarbonyl group.

**Scheme 3:**
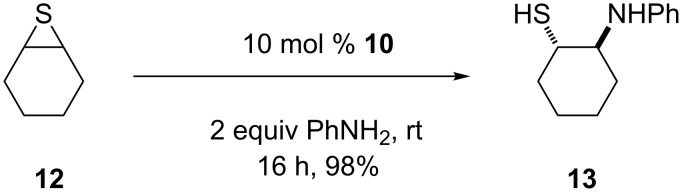
Ring opening of thiirane **12**.

Next, the behavior of these salts in the ring opening of a thiirane was explored. No activity was observed with salt **9** in the ring opening with aniline. Therefore, the more active bis-imidazolinium salt **10** was applied with thiirane **12** and aniline in CH_2_Cl_2_. A yield of 18% was obtained. However, in the absence of a solvent the yield increased to 98% after 16 h (Scheme 3). In the absence of a catalyst a yield of 12% was obtained under neat conditions. Product **13** was obtained in all reactions as racemate in the error range of the HPLC measurements.

**Scheme 4:**
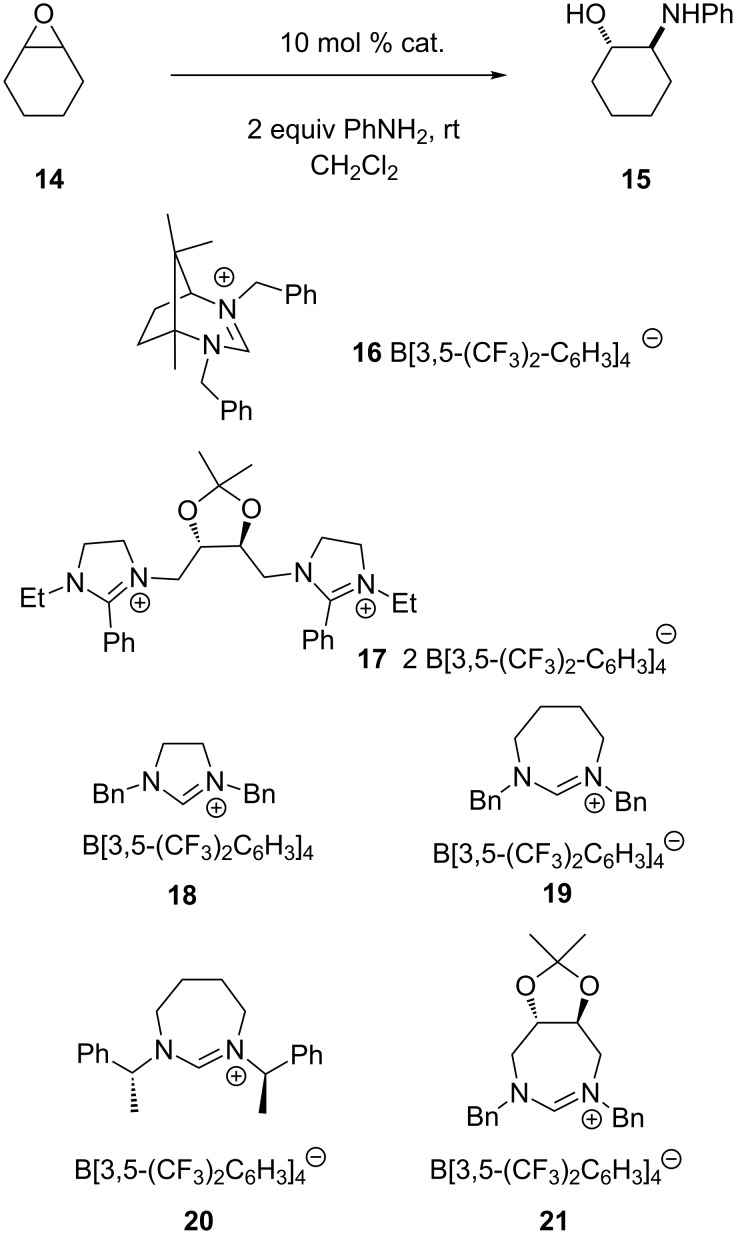
Ring opening of epoxide **14**.

Next, several salts were explored in the ring opening of cyclohexene epoxide with aniline (Scheme 4, Table 2). A reaction in CH_2_Cl_2_ in the absence of the catalyst gave a yield of 10% after 24 h (Table 2, entry 1).

The camphor based salt **16** gave a yield of 24%, while bis-imidazolinium salt **17** resulted in a yield of 60% (Table 2, entries 2 and 3). The bis-imidazolinium salts **10** and **11** gave lower yields compared to **17**. However, when the reaction was carried out in the absence of a solvent, the yield increased with salts **10** and **11** to 98 and 96%, respectively (Table 2, entries 6 and 7).

**Table 2:** Ring opening of epoxide **15** with aniline and 10 mol % salt.

entry	solvent	catalyst	time	yield %^a^

1	CH_2_Cl_2_	–	24 h	10
2	CH_2_Cl_2_	**16**	24 h	24
3	CH_2_Cl_2_	**17**	48 h	60
4	CH_2_Cl_2_	**10**	48 h	34
5	CH_2_Cl_2_	**11**	48 h	48
6	neat	**10**	24 h	98
7	neat	**11**	24 h	96
8	neat	**–**	24 h	0
9	CH_2_Cl_2_	**18**	48 h	12
10	CH_2_Cl_2_	**19**	24 h	78
11	CH_2_Cl_2_	**20**	24 h	38
12	CH_2_Cl_2_	**21**	6 h	99
13	toluene	**–**	2 h	0
14	toluene	**21**	2 h	89

^a^Isolated yields.

Compared to sulfur, oxygen is a good hydrogen-bond acceptor. Hence, also imidazolinium salts with a C(2)H unit were applied in the reaction. Here, an activation of the epoxide can be also possible through hydrogen bonding next to the direct interaction with the positively charged NCN center. While salt **18** displayed very low catalytic activity, salt **19** gave the product in 78% yield (Table 2, entry 10). This phenomenon may be explained by the better delocalization of the positive charge in the planar sp^2^-centered imidazoline scaffold in salt **18**. Meanwhile the special geometry of 7-membered 1,3-diazepinium cations such as **19** does not allow the planar conformation to be kept, thus the positive charge is less delocalized over the NCN atoms (Scheme 4) [26–29]. Salt **16**, incorporating a six-membered ring as the smallest ring, displayed a catalytic activity between salt **18** and **19**. Salt **20**, with a larger steric environment around the amidinium unit next to the nitrogen atoms, gave a yield of only 38% (Table 2, entry 11). On the other hand salt **21** gave the product in 99% yield after 6 h (Table 2, entry 12). By changing the solvent from dichloromethane to toluene the reaction time was even further reduced with salt **21**, and the product was isolated in 89% yield (Table 2, entry 14). In all cases product **15** was racemic in the error range of the HPLC measurements.

**Scheme 5:**
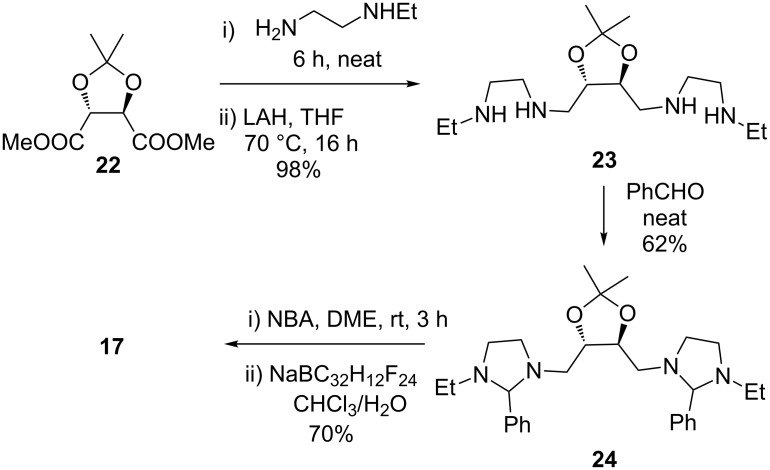
Synthesis of bis-imidazolium salt **17**.

Salt **17** was prepared according to Scheme 5. The synthetic route involved a tetraamine formation via amidation under neat conditions and reduction with LAH, furnishing the product in 98% yield over two steps. Next, the aminal formation under neat conditions was performed. The aminal was further oxidized to a bromide salt by *N*-bromoacetamide in dimethoxyethane. The bromide counter anion was further efficiently replaced by a tetrakis(3,5-bis(trifluoromethyl)phenyl)borate anion providing the salt **17** by anion metathesis.

**Scheme 6:**
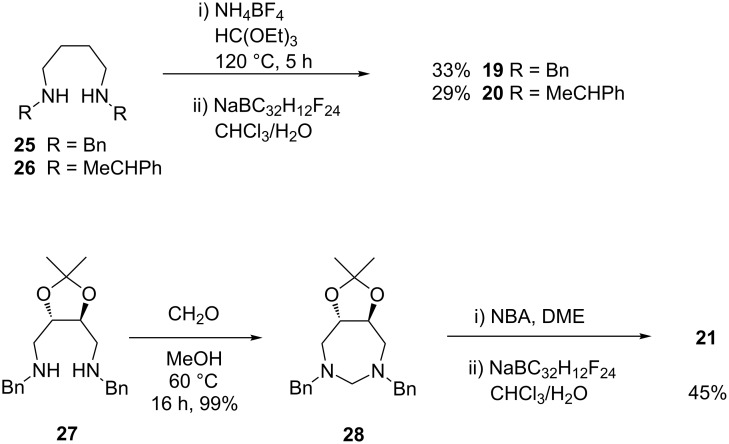
Synthesis of amidinium salt **21**.

The 7-membered 1,3-diazepinium salts were prepared according to Scheme 6. Salts **19** and **20** were prepared in a standard way by treatment with triethyl orthoformate in the presence of ammonium tetrafluoroborate followed by anion metathesis in 33 and 29% yield, respectively.

Due to the low yield, salt **21** was prepared through a different route. First amine **27** [30] was transformed with formaldehyde to the aminal **28**. The latter could be oxidized to the corresponding bromide salt, which was transformed directly by anion metathesis to the salt **21**.

## Conclusion

It was possible to prepare new metal-free Lewis acids containing bis-imidazolinium cations and investigate the salts as organocatalysts. Although with enantiopure chiral salts no enantiomeric excess was observed, it was shown for the first time that these salts can interact with thiocarbonyl groups and thiiranes in order to activate these substrates. In addition, it was found that 7-membered 1,3-diazepinium cations are good catalysts for the ring opening of epoxides and that these salts are more reactive than imidazolinium salts.

## Supporting Information

File 1 Experimental part.
