# Supplementary material for: Imidazolinium and amidinium salts as Lewis acid organocatalysts
Source: Beilstein J Org Chem. 2012 Oct 18;8:1798–803. doi: 10.3762/bjoc.8.205 (PMC3511014; doi:10.3762/bjoc.8.205)
Supplement: File 1 — Experimental part. [file Beilstein_J_Org_Chem-08-1798-s001.pdf]

# Supporting Information

**for**

## Imidazolinium and amidinium salts as Lewis acid organocatalysts

Oksana Sereda[1], Nicole Clemens[1], Tatjana Heckel[2] and René Wilhelm[1,2*]

Address: [1]Institute of Organic Chemistry, Clausthal University of Technology, 38678 Clausthal-Zellerfeld, Germany and [2]Department of Chemistry, University of Paderborn, 33098 Paderborn, Germany

Email: René Wilhelm* - rene.wilhelm@uni-paderborn.de

*Corresponding author

## Experimental part

## Experimental

### General Experimental

Salts **7** [1], **8** [1], **9** [2], **10** [2], **11** [2] and diamines **25** [3], **26** [4] and **27** [5] were prepared according to the literature.

Flash column chromatography was performed on Sorbisil C-60. All reactions were monitored by TLC with Merck Silica gel 60 $F_{254}$ plates. HRMS were recorded on Bruker Daltonik Tesla-Fourier Transform-Ion Cyclotron Resonance Mass spectrometer with Electrospray-Ionisation by Dr. Dräger at the Institute of Organic Chemistry, University of Hannover. Infrared spectra were recorded on a Perkin-Elmer 2000 FT-IR System FTIR instrument. NMR spectra were performed in $CDCl_3$ at ambient temperature on a Bruker AMX 400 and a Bruker AC 200F.

Mass spectra were recorded on Hewlett Packard 5898B (at 70 eV). Melting points were taken with an apparatus according to the method of Dr. Tottoli and are uncorrected.

## Preparation of the salts and their application as catalysts

### ***O*-Ethyl (*E*)-3-phenyl-2-propenethioate (Ethyl thionocinnamate) (5)**

(*E*)-3-Phenylacrylic acid ethyl ester (30 mL, 0.18 mol) was dissolved in anhydrous xylene (200 mL) and Lawesson's reagent (72.3 g, 0.18 mol) was added. The reaction mixture was heated under reflux for 10 h. After cooling to room temperature the reaction mixture was evaporated and applied to a column with petroleum ether/diethyl ether (95:5) as eluent to filter off the solid compounds. The concentrated residue was distilled (bp 140 °C, 16 mbar) (Lit. [6] bp 140 °C, 12 Torr). The resulting liquid was a mixture of starting material and product. These were separated by another column with pure petroleum ether as eluent. The last impurity was removed by a second distillation (bp 100 °C, 0.6 mbar) to yield **5** as an orange oily fluid (11 g, 57 mmol, 32%). IR (KBr) 3026s, 1611s, 1575s, 1495s, 1448s, 1388s, 1364s, 1039s, 970s, 752s, 689s $cm^{-1}$; $^{1}H$ NMR (200 MHz) δ 7.70 (d, *J* = 15.7 Hz, 1H), 7.59–7.54 (m, 2H, Ar-H), 7.40–7.37 (m, 3H, Ar-H), 7.02 (d, *J* = 15.9 Hz, 1H), 4.64 (q, *J* = 7.1 Hz, 2H), 1.48 (t, *J* = 7.1 Hz, 3H); $^{13}C$ NMR (50 MHz) δ 210.6, 140.5, 134.9, 130.4, 129.12, 129.09, 128.5, 68.0, 14.0. The spectral data were consistent with literature values [6].

**General procedure for the use of catalysts in the Diels–Alder reaction.** 10 mol % of the desired catalyst was dissolved in the stated dry solvent. Ethyl thionocinnamate (**5**) and 1.5 equiv freshly distilled cyclopentadiene were added and the mixture was shaken. Reaction time and reaction temperature are given in Table 1. After evaporation of the solvent the crude mixtures were purified by column chromatography on $SiO_2$ with pure petroleum ether as eluent. For results see Table 1 in the article.

**Ethyl 3-phenylbicyclo[2.2.1]hept-5-ene-2-carbothioate (6)**

Oil: MS (EI) *m*/*z*: 258 (*M*, 100%), 192 (100), 167 (50), 147 (40), 132 (75), 115 (100); $^{1}$H NMR (200 MHz) δ 7.38–7.17 (m, 5H, Ar), 6.41 (dd, *J* = 2.5 Hz, *J* = 3.1 Hz, 1H, C=C-H), 6.04 (dd, *J* = 2.5 Hz, *J* = 3.1 Hz, 1H, C=C-H), 4.49 (q, *J* = 7.2 Hz, 2H, O-$CH_2$-$CH_3$), 3.43–3.30 (m, 3H), 3.06–2.99 (m, 1H), 1.83 (d, *J* = 8.6 Hz, 1H), 1.57 (dq, *J* = 1.8 Hz, *J* = 8.6 Hz, 1H), 1.37 (t, *J* = 7.2 Hz, 3H, O-$CH_2$-$CH_3$); $^{13}$C NMR (50 MHz) δ 224.6, 144.6, 138.8, 134.0, 128.5, 127.6, 126.1, 68.3, 63.1, 49.7, 49.1, 48.9, 47.6, 13.8; HPLC (OD-H): Detector 254 nm, Flow 0.1 mL/min, hexane, retention time $t_{R1}$ = 52 min, $t_{R2}$ = 55 min.

***trans*-2-(Phenylamino)cyclohexanethiol (13)**

1 Equiv cyclohexane sulfide (0.3 mmol, 34 mg) was added to a mixture of 2 equiv aniline (0.6 mmol, 55 μL) and 0.1 equiv catalyst (0.03 mmol, 43.2 mg). After being stirred for over 16 h at rt, the reaction mixture was submitted to flash column chromatography on silica gel with ethyl acetate/petroleum ether (1:9) mixture to give the product in 98% yield as a colorless oil. The spectral data were consistent with literature values [7].

**General procedure for the use of catalysts in the ring opening of cyclohexene epoxide (14).** To a mixture of 2 equiv aniline (0.4 mmol) and 0.1 equiv catalyst (0.02 mmol) in toluene (2 mL) or $CH_2Cl_2$ (1.5 mL), 1 equiv epoxide (0.2 mmol) was added. The mixture was stirred for 3–72 h at rt. The reaction mixture was directly submitted to flash column chromatography on silica gel with ethyl acetate/petroleum ether (1:8) mixture to give the products as colorless oils. The catalysts were eluted from the column with $CH_2Cl_2$. The spectral data were consistent with literature values [8]. For results see Table 2 in the article.

**(1*S*,5*S*)-2,4-Dibenzyl-5,8,8-trimethyl-4-aza-2-azoniabicyclo[3.2.1]oct-2-ene tetrakis(3,5-bis(trifluoromethyl)phenyl)borate (16)**

From (1*S*,5*S*)-2,4-dibenzyl-5,8,8-trimethyl-4-aza-2-azoniabicyclo[3.2.1]oct-2-ene tetrafluoroborate [9] (0.125 g, 0.68 mmol) and sodium tetrakis(3,5-bis(trifluoromethyl)phenyl)borate (0.26 g, 0.30 mmol) after being stirred at rt in $CH_2Cl_2$ (2 mL) and $H_2O$ (2 mL) for 30 min to give the product as a light yellow oil in 42 % yield (0.34 g, 0.29 mmol). $[\alpha]^{20}_{D}$ = +59 (*c* 0.56, $CHCl_3$); MS (ESI) *m*/*z*: 333 $[M]^+$; IR (KBr) 3072, 3041, 2991, 1665, 1612, 1471, 1459, 1391, 1356, 1279 $cm^{-1}$; $^1$H-NMR (200 MHz) δ 7.93 (s, 1H, NC*H*N), 7.71 (s, 8H, *H*-($CF_3$)Ar), 7.53 (s, 4H, *H*-($CF_3$)Ar), 7.38–7.33 (m, 6H, *H*-Ar), 7.10–7.02 (m, 4H, *H*-Ar), 4.39 (s, 2H, C$H_2$C$H_2$N), 4.30 (d, *J* = 5 Hz, 2H, C$H_2$N), 3.18 (d, *J* = 4 Hz, 1H, C$H_2$N), 2.32–2.16 (m, 1H, C$H_2$), 2.07–1.82 (m, 3H, C$H_2$), 1.30 (s, 3H, ($CH_3$)$_2$C), 1.00 (s, 3H, C*H*3C), 0.83 (s, 3H, ($CH_3$)$_2$C); $^{13}$C NMR (50 MHz) δ 161.7 (q, *J* = 49.7 Hz), 150.8, 134.8, 131.5, 130.3, 130.2, 130.1, 130.0, 129.8, 129.20 (q, *J* = 2.85 Hz, $CF_3$-*C*Ar), 128.9, 128.6 (q, *J* = 3 Hz, $CF_3$-*C*Ar), 128.3, 124.5 (q, *J* = 270 Hz, *C*$F_3$), 117.5 (q, *J* = 4.1 Hz, $CF_3$*C*H$CF_3$), 72.2, 67.4, 58.3, 53.7, 40.7, 39.6, 31.5, 21.1, 16.6, 14.3; HRMS: Calcd for $C_{23}H_{29}N_2$, 333.2331 $[M]^+$; found, 333.2327.

**3,3'-((4S,5S)-2,2-Dimethyl-1,3-dioxolane-4,5-diyl)bis(methylene)bis(1-ethyl-2-phenylimidazolidinium) di(tetrakis(3,5-bis(trifluoromethyl)phenyl)borate) (17)**

*3,3'-((4S,5S)-2,2-Dimethyl-1,3-dioxolane-4,5-diyl)bis(methylene)bis(1-ethyl-2-phenylimidazolidinium) dibromide:* The 1 equiv diaminal **24** (0.35 g, 0.73 mmol) was dissolved in DME (1 mL), and the solution was cooled on the ice bath to 0 °C. *N*-Bromoacetamide (0.212 g, 1.54 mmol) was next added in portions. After being stirred at rt for 3 h, the oil separated and DME was carefully decanted off. The remaining brown oil was washed with petrol ether and diethyl ether and was dried in vacuo to provide the dibromide

salt as a highly hygroscopic brown solid in a crude yield of 95% (0.438 g, 0.69 mmol). The salt was used in the next step without purification.

*3,3'-((4S,5S)-2,2-Dimethyl-1,3-dioxolane-4,5-diyl)bis(methylene)bis(1-ethyl-2-phenylimidazolidinium) di(tetrakis(3,5-bis(trifluoromethyl)phenyl)borate) (**17**)*: The bromide salt was dissolved in $CHCl_3$ and a solution of sodium tetrakis(3,5-bis(trifluoromethyl)phenyl)borate in water was added. The mixture was stirred for 45 min. Then the organic phase was separated, washed with brine, dried ($Na_2SO_4$) and concentrated. The salt was purified by flash column chromatography on silica gel with 2.5% methanol in $CH_2Cl_2$ to give a light yellow oil in 74% yield. $[\alpha]^{20}_D$ = –8.4 (*c* 1.68, $CH_3OH$); MS (ESI) *m/z*: 238 $[M/2]^+$; IR (NaCl, neat): 1278, 1123, $cm^{-1}$. $^1H$ NMR (200 MHz, $CD_3OD$) δ 7.70–7.51 (m, 32H, *H*-Ar), 7.46–7.42 (m, 2H, *H*-Ar), 4.11–4.04 (m, 6H, $CH_2N$), 3.90–3.87 (m, 2H, C*H*C), 3.32–3.18 (m, 10H, Et-$CH_2$, $CH_2N$), 1.27 (s, 6H, $CH_3$), 1.15 (t, *J* = 7.2 Hz, 6H, Et-$CH_3$); $^{13}C$ NMR (50 MHz, $CD_3OD$) δ 168.1, 162.9 (q, *J* = 49 Hz), 135.9, 134.3, 131.0, 130.8 (q, *J* = 2.8 Hz), 130.2, 129.1, 125.8 (q, *J* = 270 Hz), 123.3, 118.5, 112.7, 76.6, 50.9, 49.6, 44.0, 27.2, 12.9; HRMS: Calcd for $C_{29}H_{40}N_4O_2$, 238.1556 $[M/2]^+$; found, 238.1570.

**1,3-Dibenzyl-4,5,6,7-tetrahydro-1*H*-1,3-diazepin-3-ium tetrakis(3,5-bis(trifluoromethyl)-phenyl)borate (19)**

*1,3-Dibenzyl-4,5,6,7-tetrahydro-1,3-diazepin-1-ium tetrafluoroborate*: *N,N*-Dibenzylbutylenediamine (**25**) (0.85 g, 3.16 mmol, 1 equiv), triethyl orthoformate (0.53 mmol, 3.16 mmol, 1 equiv) and ammonium tetrafluoroborate (331 mg, 3.16 mmol, 1 equiv) were heated for 5 h at 120 °C in a closed vessel. Recrystallization in dry ethanol gave a white solid (1.02 g, 2.65 mmol, 84 %). Mp 122 °C; MS (ESI, 0 V) *m/z*: 279 ($M^+$, 100 %); IR (KBr) 3029, 1675, 1471, 1452, 1359, 1241, $cm^{-1}$; $^1H$ NMR (200 MHz) δ 8.36 (s, 1H, NCHN), 7.36 (m, 10H, H-Ar), 4.68 (s, 4H, $NCH_2Ar$), 3.56 (t, *J* = 5.6 Hz, 4H, 2 $NCH_2CH_2$), 1.79 (m,

4H, 2 $NCH_2CH_2$); $^{13}$C NMR (50 MHz) δ 158.6, 133.6, 129.4, 129.1, 128.8, 61.6, 49.1, 24.6; HRMS: Calcd for $C_{19}H_{23}N_2^+$, 279.1856 $[M]^+$; found, 279.1861.

*1,3-Dibenzyl-4,5,6,7-tetrahydro-1H-1,3-diazepin-3-ium tetrakis(3,5-bis(trifluoromethyl)-phenyl)borate (**19**)*: From 1,3-dibenzyl-4,5,6,7-tetrahydro-1*H*-1,3-diazepin-3-ium tetrafluoroborate (0.125 g, 0.683 mmol), sodium tetrakis(3,5-bis(trifluoromethyl)phenyl)-borate (0.303 g, 0.683 mmol) after being stirred at rt in $CHCl_3$ (2 mL) and $H_2O$ (2 mL) for 30 min to give the product as a light yellow oil, which solidified upon standing, in 47 % (0.37 g, 0.32 mmol) yield. MS (ESI) *m*/*z*: 279 $[M]^+$; IR (KBr) 3039, 2963, 1684, 1612, 1470, 1359 $cm^{-1}$; $^1$H NMR (200 MHz) δ 7.92 (s, 1H, NC*H*N), 7.71 (s, 8H, *H*-Ar(F)), 7.52 (s, 4H, *H*-Ar(F)), 7.44–7.36 (m, 6H, *H*-Ar), 7.12–7.08 (m, 4H, *H*-Ar), 4.39 (s, 4H, PhC$H_2$N), 3.56–3.50 (m, 4H, C$H_2$N), 1.85–1.79 (m, 4H, C$H_2$CH$_2$); $^{13}$C NMR (50 MHz) δ 160.2, 155.7, 134.8, 130.9, 130.2, 129.9, 129.2 (q, $J$ = 2.55 Hz), 128.6 (q, $J$ = 1.9 Hz), 127.9, 124.5 (q, $J$ = 270.7 Hz), 117.5 (q, $J$ = 4.55 Hz), 62.1, 50.4, 24.2; HRMS: Calcd for $C_{19}H_{23}N_2^+$, 279.1856 $[M]^+$; found, 279.1862.

**1,3-Bis((*R*)-1-phenylethyl)-4,5,6,7-tetrahydro-1*H*-1,3-diazepin-3-ium tetrakis(3,5-bis(trifluoromethyl)phenyl)borate (20)**

*1,3-Bis((R)-1-phenylethyl)-4,5,6,7-tetrahydro-1H-1,3-diazepin-3-ium tetrafluoroborate*: From (*N*,*N*)-bis((*R*)-1-phenylethyl)butane-1,4-diamine (**26**) (0.39 g, 1.32 mmol), ammonium tetrafluoroborate (0.084 g, 1.01 mmol) and triethyl orthoformate (1.1 mL, 6.62 mmol) in *o*-dichlorobenzene (1 mL) upon heating at 120 °C for 5 h to give 29% yield of the product as a white oily solid. The salt was recrystallized from chloroform. $[\alpha]^{20}_D$ = +59 (*c* 0.56, $CHCl_3$). MS (ESI) *m*/*z*: 307 $[M]^+$; IR (KBr) 3033, 2991, 1658, 1580, 1493, 1451, 1368 $cm^{-1}$; $^1$H NMR (200 MHz) δ 8.36 (s, 1H, NC*H*N), 7.39–7.35 (m, 10H, *H*-Ar), 7.05–6.99 (m, 4H, *H*-Ar), 5.12 (q, $J$ = 6.8 Hz, 2H, $CH_3$C*H*N), 3.55–3.38 (m, 4H, C$H_2$N), 1.76–1.70 (m, 10H, C$H_3$CH,

C$H_2$C$H_2$); $^{13}$C NMR (50 MHz) δ 155.7, 136.1, 128.2, 127.9, 126.3, 64.0, 43.9, 23.6, 16.6; HRMS: Calcd for $C_{21}H_{27}N_2$, 307.2174 $[M]^+$; found, 307.2174.

*1,3-Bis((R)-1-phenylethyl)-4,5,6,7-tetrahydro-1H-1,3-diazepin-3-ium tetrakis(3,5-bis(trifluoromethyl)phenyl)borate (**20**)*: From 1,3-bis((*R*)-1-phenylethyl)-4,5,6,7-tetrahydro-1*H*-1,3-diazepin-3-ium tetrafluoroborate (0.246 g, 0.625 mmol), sodium tetrakis(3,5-bis(trifluoromethyl)phenyl)borate (0.554 g, 0.625mmol) after being stirred at rt in $CHCl_3$ (5 mL) and $H_2O$ (2 mL) for 30 min to give the product as a light oil in 29% (0.22 g, 0.187 mmol) yield. $[\alpha]^{20}_D$ = +18 (*c* 0.26, $CHCl_3$); MS (ESI) *m*/*z*: 279 $[M]^+$; IR (KBr) 3020, 1355, 1280 $cm^{-1}$; $^1$H NMR (200 MHz) δ 7.71 (s, 8H, *H*-Ar), 7.52 (s, 4H, *H*-Ar), 7.41–7.38 (m, 6H, *H*-Ph), 7.14–7.09 (m, 4H, *H*-Ph), 4.61 (q, *J* = 6.8 Hz, 2H,$CH_3$C*H*N), 3.52 (t, *J* = 5.4 Hz, 4H, C$H_2$N), 1.86–1.77 (m, 4H, C$H_2$C$H_2$), 1.59 (d, *J* = 6.8 Hz, 6H, C$H_3$); $^{13}$C NMR (50 MHz) δ 163.1, 153.9, 135.3, 134.7, 130.1, 129.9, 129.2 (q, *J* = 3.1 Hz), 128.6 (q, *J* = 2.8 Hz), 126.6, 124.5 (q, *J* = 270.8 Hz), 117.4 (q, *J* = 3.4 Hz), 76.4, 47.8, 24.4, 18.2; HRMS: Calcd for $C_{21}H_{27}N_2^+$, 307.2174 $[M]^+$; found, 307.2174.

**(3a*S*,8a*S*)-5,7-dibenzyl-2,2-dimethyl-4,7,8,8a-tetrahydro-3a*H*-[1,3]dioxolo[4,5-e][1,3]diazepin-5-ium tetrakis(3,5-bis(trifluoromethyl)phenyl)borate (21)**

*(3aS,8aS)-5,7-Dibenzyl-2,2-dimethylhexahydro-3aH-[1,3]dioxolo[4,5-e][1,3]diazepine (**28**)*: The dioxolane diamine **27** (0.773 g, 2.27 mmol) was dissolved in methanol (45 mL) and was treated with an aqueous formaldehyde solution (0.3 mL, 3.39 mmol). The reaction mixture was stirred in a sealed flask overnight. After completion of the reaction, the volatiles were removed by rotary evaporation. The crude product was dissolved in $CH_2Cl_2$, and dried ($Na_2SO_4$). The solution was concentrated and the product was dried in vacuo to furnish the aminal as a yellow viscous oil in 99% (0.80 g, 2.26 mmol) yield. The aminal was used in the next step as such.

*(3aS,8aS)-5,7-Dibenzyl-2,2-dimethyl-4,7,8,8a-tetrahydro-3aH-[1,3]dioxolo[4,5-e][1,3]diazepin-5-ium bromide*: The solution of 1 equiv aminal **28** (0.796 g, 2.26 mmol) in dimethoxyethane (1 mL) was cooled to 0 °C, and 1 equiv NBA (0.312 g, 2.26 mmol) was added portion wise. After addition the mixture was stirred for 3 h at rt. The product was precipitated from the reaction mixture by addition of diethyl ether. The crude salt was recrystallized from methanol/diethyl ether and finally washed with diethyl ether to afford the product as a brown oil in 82% (1.25 g, 1.85 mmol) yield.

*(3aS,8aS)-5,7-Dibenzyl-2,2-dimethyl-4,7,8,8a-tetrahydro-3aH-[1,3]dioxolo[4,5-e][1,3]diazepin-5-ium tetrakis(3,5-bis(trifluoromethyl)phenyl)borate (**21**)*: The mixture of 1 equiv bromide salt (0.55g, 1.20 mmol) and 1 equiv sodium tetrakis(3,5-bis(trifluoromethyl)phenyl)borate (1.06 g, 1.20 mmol) in $CH_2Cl_2$/water mixture was stirring vigorously at rt for 3 h. The mixture was concentrated, and the residue was extracted with $CHCl_3$. The extracts were washed once with brine, dried over 3 Å MS and concentrated to furnish the product as a yellow oil. The salt was purified by FCC on silica gel with $CH_2Cl_2$. Yield: 45% (0.66 g, 0.54 mmol). $[\alpha]^{20}_{D}$ = –3.8 (*c* 0.53, $CHCl_3$); MS (ESI) *m/z*: 331 [M + H]; IR (NaCl, $CHCl_3$) 2969, 1654, 1533, 1377, 1215 $cm^{-1}$. $^{1}H$ NMR (400 MHz) δ 7.76 (s, 8H, *H*-Ar(F)), 7.68 (s, 1H, NC*H*N), 7.52 (s, 4H, *H*-Ar(F)), 7.48–7.40 (m, 8H, *H*-Ar), 7.12 (s, 2H, *H*-Ar), 7.10 (s, 2H, *H*-Ar), 4.52 (s, 4H, $CH_2N$), 3.87–3.80 (m, 4H, $CH_2N$), 3.41–3.32 (m, 2H, $CH_2CH$), 1.40 (s, 6H, $(CH_3)_2C$); $^{13}C$ NMR (100 MHz) δ 161.7 (q, *J* = 49.8), 154.7, 134.8, 132.5, 130.8, 130.2, 129.1 (q, *J* = 2.8 Hz), 128.8 (q, *J* = 2.7 Hz), 128.2, 124.6 (q, *J* = 270.9 Hz), 117.6 (d, *J* = 4.1 Hz), 114.2, 74.9, 65.1, 53.1, 26.5; HRMS: Calcd for $C_{22}H_{27}N_2O_2^{+}$, 351.2073 $[M]^{+}$; found, 351.2087.

***N*,*N*-((4*S*,5*S*)-2,2-dimethyl-1,3-dioxolane-4,5-diyl)bis(methylene)bis(*N*2-ethylethane-1,2-diamine) (23)**

*(4R,5R)-N4,N5-Bis(2-(ethylamino)ethyl)-2,2-dimethyl-1,3-dioxolane-4,5-dicarboxamide:*
1 Equiv (–)-dimethyl-2,3-*O*-isopropylidene-L-tartrate (**22**) (0.2 mL, 1 mmol) was added to an excess of *N*-ethylethylenediamine (1 mL, 9 mmol) dropwise under nitrogen over 45 min. After the stirring at rt for 6 h, the mixture was applied to vacuum, and the excess of the amine was removed. No further purification was necessary, and the product was furnished as yellow oil in 99% (0.33 g, 0.996 mmol) yield. $[\alpha]^{20}_{D}$ = –3.8 (*c* 0.53, $CHCl_3$); MS (ESI) *m*/*z*: 331 $[M + H]^+$; IR (NaCl, neat) 3300, 2969, 1654, 1533, 1377 $cm^{-1}$; $^1H$ NMR (200 MHz) δ 7.34 (bs, 2H, CON*H*), 4.55 (s, 2H, C*H*OC), 3.41 (d, *J* = 5.8 Hz, 2H, C$H_2$N), 3.35 (d, *J* = 5.8 Hz, 2H, C$H_2$N), 2.77 (t, *J* = 6 Hz, 4H, C$H_2$N), 2.64 (q, *J* = 7 Hz, 4H, Et-C$H_2$), 1.45 (s, 6H, C$H_3$), 1.22 (bs, 2H, N*H*), 1.08 (t, *J* = 7 Hz, 6H, Et-C$H_3$); $^{13}C$ NMR (50 MHz) δ 169.8, 112.3, 77.6, 48.3, 43.6, 39.0, 26.1, 15.3; HRMS: Calcd for $C_{15}H_{31}N_4O_4$, 331.2345 $[M + H]^+$; found, 331.2345.

*N,N-((4S,5S)-2,2-dimethyl-1,3-dioxolane-4,5-diyl)bis(methylene)bis(N2-ethylethane-1,2-diamine) (**23**):* The solution of (4*R*,5*R*)-*N*4,*N*5-bis(2-(ethylamino)ethyl)-2,2-dimethyl-1,3-dioxolane-4,5-dicarboxamide (0.33 g, 1 mmol) in dry THF (4 mL) was added to the suspension of LAH (0.13 g, 3.5 mmol) under reflux in dry THF (13 mL). After being heated under reflux for 16 h, the reaction mixture was cooled down to rt and quenched carefully with water (0.2 mL) and 4 M $NaOH_{(aq)}$ (0.8 mL), followed by water (0.4 mL). The precipitate formed was filtered off and washed with $CH_2Cl_2$. The product was extracted from the filtrate with $CH_2Cl_2$. The combined organic phases were washed with sat. $Na_2CO_{3(aq)}$ and dried ($Na_2SO_4$), and the solvent was removed by rotary evaporation to give the corresponding product in 98% yield as a light yellow oil. $[\alpha]^{20}_{D}$ = –6 (*c* 0.65, $CHCl_3$); MS (ESI) *m*/*z*: 303 $[M + H]^+$; IR (NaCl, neat) 3303, 2969, 1695, 1520, 1383 $cm^{-1}$; $^1H$ NMR (200 MHz) δ 3.87–

3.83 (m, 2H, C*H*OC), 2.75–2.65 (m, 12H, C$H_2$N), 2.61 (q, *J* = 7 Hz, 4H, Et-C$H_2$), 1.87 (bs, 4H, N*H*), 1.35 (s, 6H, C$H_3$), 1.07 (t, *J* = 7 Hz, 6H, Et-C$H_3$); $^{13}$C NMR (50 MHz) δ 108.8, 78.8, 52.1, 49.6, 49.1, 44.0, 27.2, 15.2; HRMS: Calcd for $C_{15}H_{35}N_4O_2$, 303.2760 $[M + H]^+$; found, 303.2761.

**3,3'-((4*S*,5*S*)-2,2-Dimethyl-1,3-dioxolane-4,5-diyl)bis(methylene)bis(1-ethyl-2-phenylimidazolidine) (24)**

To the suspension of 1 equiv diamine (0.505 g, 1.67 mmol) in water (2.5 mL), 2 equiv benzaldehyde (0.178 g, 3.35 mmol) was added in one portion. The mixture was vigorously stirred for 5 h, after which it was diluted with $CHCl_3$ and extracted (3 × 4 mL $CHCl_3$). The combined organic fractions were dried ($Na_2SO_4$), and the crude aminal was treated with boiling hexane. The clear solution was carefully decanted from the brownish oily residue, and the rest was once more extracted with boiling hexane. On cooling, the hexane solution furnished a light yellow oil in 62% (0.495 g, 1.03 mmol) yield. The bis-aminal was used directly in the next step.

## References


1. Clemens, N.; Sereda, O.; Wilhelm, R. *Org. Biomol. Chem.* **2006,** *4*, 2285.
2. Jurčík, V.; Wilhelm, R. *Tetrahedron: Asymmetry* **2006**, *17*, 801.
3. Ruiz-Gomez, G.; Iglesias, M. J.; Serrano-Ruiz, M.; Garcia-Granada, S.; Franceschi, A.; Lopez-Ortiz, F.; Cuevas, C. *J. Org. Chem.* **2007**, *72*, 3790.
4. Mastrano, V. M.; Quintero, L.; de Parrodi, C. A.; Juaristi, E.; Walsh, P. J. *Tetrahedron* **2004**, *8*, 1781.
5. Angelovski, G.; Keraenen, M. D.; Eilbracht, P. *Tetrahedron: Asymmetry* **2005**, *16*, 1919.

6. Alper H.; Brandes, D. A. *Organometallics* **1991**, *10*, 2457.

7. Mishra, A.; Ali, A.; Upreti, S.; Whittingham, M. S.; Gupta, R. *Inorg. Chem.* **2009**, *48,* 5234.

8 Hou, X.-L-; Wu, J.; Dai, L.X.; Xia, L.-J.; Tang, M.-H. *Tetrahedron:Asymmetry* **1998**, *9*, 1747.

9 Peddiahgari, V. G. R.; Tabassum, S.; Blanrue, A.; Wilhelm, R. *Chem. Commun.* **2009**, 5910.
